# Can Preoperative 3D Printing Change Surgeon's Operative Plan for Distal Tibia Fracture?

**DOI:** 10.1155/2019/7059413

**Published:** 2019-02-11

**Authors:** Hwa Jun Kang, Bom Soo Kim, Seung Min Kim, Yu Mi Kim, Hyong Nyun Kim, Jae Yong Park, Jae Ho Cho, Youngrak Choi

**Affiliations:** ^1^Department of Orthopedic Surgery, Hallym Sacred Heart Hospital, Hallym University College of Medicine, Anyang-si 896, Republic of Korea; ^2^Department of Orthopedic Surgery, Inha University Hospital, Incheon 7-206, Republic of Korea; ^3^Department of Orthopedic Surgery, Sanbon Hospital, Wonkwang University College of Medicine, Gunpo-si 1126-1, Republic of Korea; ^4^Department of Orthopedic Surgery, Kangnam Sacred Heart Hospital, Hallym University College of Medicine, Seoul 948-1, Republic of Korea; ^5^Department of Orthopedic Surgery, Chuncheon Sacred Heart Hospital, Hallym University College of Medicine, Chuncheon-si 153, Republic of Korea; ^6^Department of Orthopedic Surgery, CHA Bundang Medical Center, CHA University, Seongnam-si 351, Republic of Korea

## Abstract

This study aimed to determine if 3D printing can affect surgeon's selection of plate for distal tibia fracture surgery and to find out whether orthopedic surgeons consider this technology necessary and would use it in their practice. A total of 102 orthopedic surgeons were asked to choose anatomically contoured locking plates among 5 most commonly used types for one simple and one complex distal tibia fracture based on X-ray and CT images. Next, they were provided real-size 3D printed models of the same fractures, allowed to apply each of the 5 plates to these models, and asked if they would change their choice of plate. A 10-point numeric rating scale was provided to measure the extent of the help that 3D printing provided on preoperative planning. Finally, we asked the surgeons if they would use 3D printing in their practice. Seventy-four percent of inexperienced surgeons changed their selection of plate after using 3D printed models for the complex fracture. In contrast, only 9% of experienced surgeons changed their selection of plate for the simple fracture. Surgeons rated the extent of usefulness of the 3D models in preoperative planning as a mean of 4.84 ± 2.54 points for the simple fracture and 6.63 ± 2.54 points for the complex fracture. The difference was significant (p < 0.001). Eighty-six percent of inexperienced surgeons wanted to use 3D models for complex fractures. However, only 18% of experienced surgeons wanted to use 3D printed models for simple fractures. The use of a real-size 3D-printed model often changed surgeon's preoperative selection of locking plates, especially when inexperienced surgeons evaluated a complex fracture. However, experienced surgeons did not find 3D models very useful when assessing simple fractures. Future applications of 3D models should focus on training beginners in fracture surgery, especially when complex fractures are concerned.

## 1. Introduction

Anatomically contoured locking plates are most commonly used in the treatment of distal tibia fracture to facilitate minimally invasive plate osteosynthesis (MIPO) [1–5]. Various plates from different manufacturers are commercially available [6]. However, relatively thin soft tissue coverage over the distal tibia can result in plate prominence and skin-related complications [2]. Mismatch between the plate and the fractured bone can result in inadequate fixation stability. In a biomechanical study, increased distance between the locking plate and the bone significantly affected construct stability [7]. It was recommended that the plate should be placed at a distance less than 2 mm from the bone [7]. Although plate bending can improve the fit, most anatomically contoured plates are made of difficult to bend materials such as titanium alloy and stainless steel, and the substantial force required for bending can lower the tensile strength of the plate. Furthermore, for many locking plates, screws are inserted at a predetermined fixed angle that cannot be adjusted according to the fracture pattern or fragment direction during the installation. Therefore, the choice of plate for MIPO is important for successful implantation. In the process of selection of an appropriate anatomically contoured locking plate, three-dimensional (3D) printing may be useful for identification of the best-fitting plate and may also help select a locking plate with optimal locking screw trajectories, such that the inserted screws enhance stability [8–10].

Three-dimensional printing enables reproduction of the actual osseous anatomy via a real-size model to which locking plates of different kinds can be applied preoperatively to identify the best-fitting plate [8–16]. The purpose of this study was to determine if 3D printing can affect the surgeon's selection of the plate for distal tibia fracture surgery and to find out if orthopedic surgeons find this technology sufficiently useful in distal tibia fracture for it to be actually employed in clinical practice.

We hypothesized that simulating the plate attachment to the fractured bone using a real-size 3D printed model would shift the choice of locking plates towards the ones providing the best fit and that orthopedic surgeons would find 3D printing sufficiently useful in MIPO of distal tibia fracture for it to be utilized in their practice.

## 2. Materials and Methods

### 2.1. Preparation to Interviews

**Figure 1:**
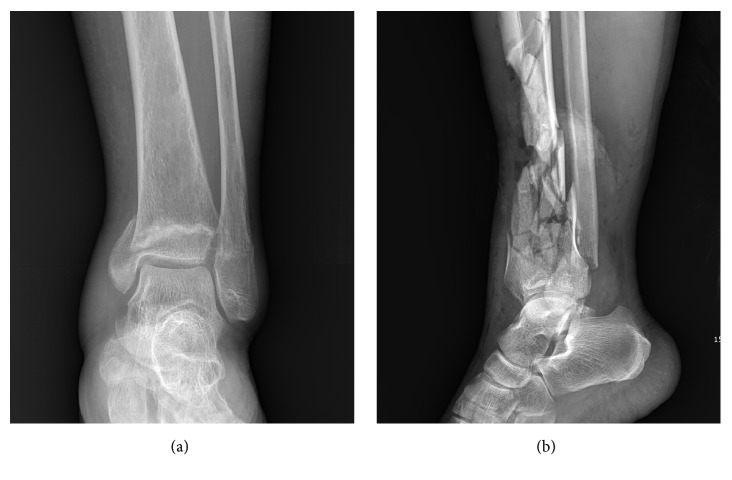
Two cases of distal tibia fractures, one simple (a) and one complex (b), were chosen for the study.

This study used one-on-one interviews to explore orthopedic surgeons' views on the use of 3D printing in distal tibia fracture surgery. Institutional review board approval was obtained for the study. Two cases of distal tibia fracture (one simple and one complex) were chosen (Figure 1).

After obtaining informed consent, corresponding X-ray (AP, lateral, mortise views) and CT (sagittal, coronal, axial, and 3D reconstructed images) images were extracted from the PACS (Picture Archiving and Communication System) and stored in a tablet PC to provide the interviewees with radiological images of the fractured bone during the interview.

**Figure 2:**
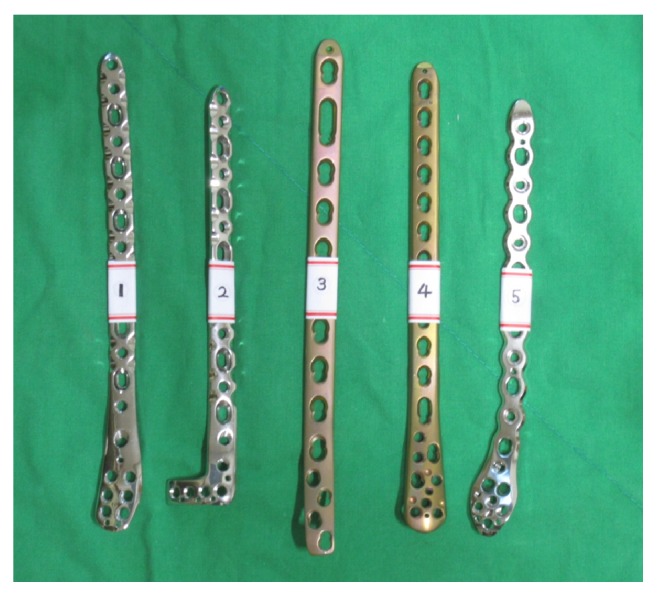
A picture of the 5 most commonly used anatomically designed locking plates for MIPO of distal tibia fracture was shown to the interviewees and asked which plate they will use for each of the two fractures.

A picture of the 5 most commonly used anatomically designed locking plates for MIPO of distal tibia fracture was prepared (Figure 2).

**Figure 3:**
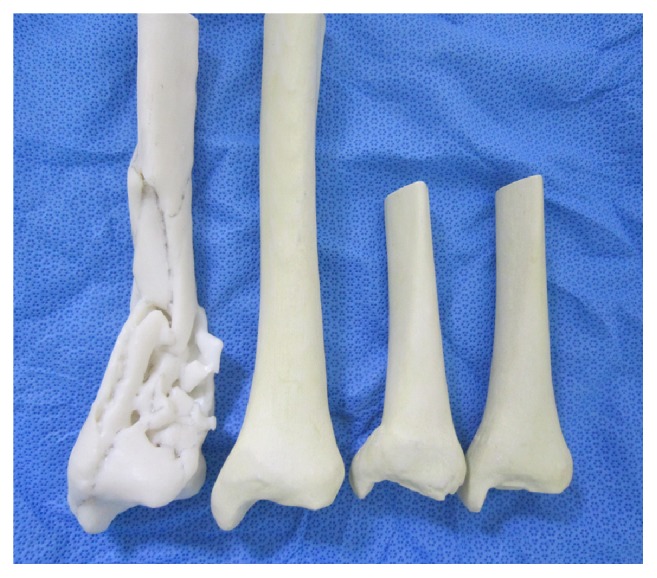
Then, surgeons were provided with real-size 3D models of the fractured tibia and the normal tibia created by mirroring the tibia on the unaffected side before 3D printing.

Technical guides and brochures provided by the manufacturer were also prepared for each plate to provide the interviewee with the necessary information. A survey sheet with 5 questions was developed. A supplementary material file shows this in more detail (see Supplementary Material File 1). The initial version of this survey sheet was examined by 6 consultants, and feedback on contents and ease of use was obtained. Five different most commonly used anatomically designed locking plates were prepared using a locking drill sleeve to show locking screw trajectories for each screw hole. Two real-size 3D printed models were prepared, one of the fractured bone and one of the normal bone on the opposite side, which was mirrored during 3D printing to simulate the preinjury tibia (Figure 3).

### 2.2. 3D Printing of Real-Size Tibia Models

**Figure 4:**
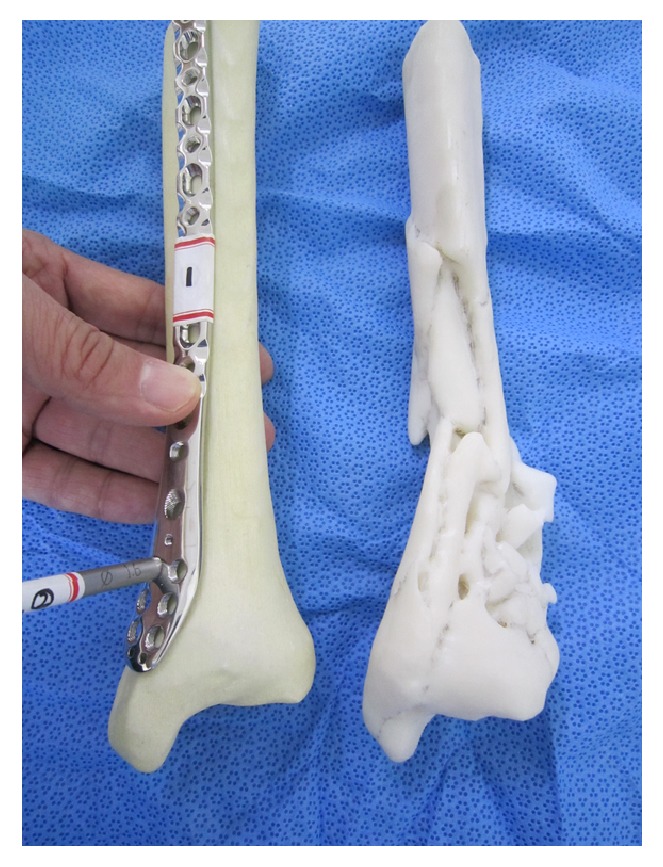
Surgeons were given time to use these 3D models to study the fracture configuration and to simulate the placement of the plates on the fractured tibia considering screw trajectories in the plate for fixation of fracture fragments. The normal-side tibia model was provided to simulate the fractured tibia after reduction. Surgeons were then asked again to select the plates most suitable for the two distal tibia fractures.

After obtaining informed consent, CT data of two patients with distal tibia fracture were used to produce real-size 3D tibia models. Specialized software (MIMICS: Materialise Interactive Medical Image Control System Software, Materialise, Leuven, Belgium) was used to convert the CT data, which were stored in the DICOM (Digital Imaging and Communications in Medicine) format, to a standard triangulation language file format recognized by the 3D printer. A real-size fractured tibia model was printed with an inkjet printing technique using a 3D printer (Projet x60 series, 3D System Inc., Rock Hill, SC, USA). A suitable replica of the same tibia before the injury was created by mirroring the tibia on the other side before 3D printing (Figure 4).

### 2.3. Subjects and Interview Administration

A total of 102 orthopedic surgeons were interviewed. Among them, 24 were foot and ankle consultants and professors working at university hospitals and 25 were board certified orthopedic surgeons working at local hospitals or clinical fellows specializing in foot and ankle surgery. Fifty-three surgeons were residents working at eight university hospitals. Forty-five interviewees served as the operating surgeon during surgery for distal tibia fracture in more than 15 cases (defined as the experienced group), whereas the remaining 57 surgeons had less than 15 cases of such experience (defined as the inexperienced group). During the interview, surgeons were first shown X-ray (AP, lateral, mortise views) and CT (sagittal, coronal, axial, and 3D reconstructed images) images of the distal tibia fracture on a tablet PC (Figure 2). One case of distal tibia fracture was a simple fracture, and the other case was a complex fracture. A picture of five most commonly used anatomically designed contoured locking plates for MIPO of distal tibia fracture was shown to the surgeons (Figure 3). Technical guides and brochures for each plate were provided. Surgeons were then given a survey sheet with the first question. The first question was “Which plates would you use for each of the two distal tibia fractures?" Surgeons were given time to complete the first question. The survey sheet contained 6 options to choose from. Five options corresponded to the 5 locking plates on the picture provided, and the 6th option was to select an implant not present in the picture [17, 18].

Then, surgeons were provided with real-size 3D models of the fractured tibia and the normal tibia created by mirroring the tibia on the unaffected side before 3D printing (Figure 4). Surgeons were given time to use these 3D models to study the fracture configuration and to simulate the placement of the plates on the fractured tibia considering screw trajectories in the plate for fixation of fracture fragments. The normal-side tibia model was provided to simulate the fractured tibia after reduction (Figure 4).

The fractured models and mirrored normal-side models were provided to help surgeons select the plate with the best fit and optimal configuration of locking screw holes, such that the locking screw trajectories after insertion would enhance plate stability in the best possible way (Figure 4). Surgeons were then asked again to select the plates most suitable for the two distal tibia fractures. The third question was “How useful was 3D printing in evaluating fracture configuration?" A 10-point numerical rating scale (NRS; 0 for not useful at all; 10 for extremely useful) was used to measure the extent of usefulness of 3D printing in assessing each tibia fracture. A 10-point NRS was included in the survey sheet for the third question. The fourth question was "How useful was 3D printing in preoperative planning and selection of the locking plate?" A 10-point NRS was included for each tibia fracture. Finally, surgeons were asked if they would use 3D models in their practice for distal tibia fracture if such models were available.

### 2.4. Statistical Analysis

Statistical analysis was performed using SPSS, version 23.0 (SPSS, SPSS Inc., Chicago, IL, USA). Data normality was assessed by the Kolmogorov–Smirnov test. The Chi-square test was used to determine if changes of plate selection after use of 3D printed models were significantly related to complexity of tibia fracture (simple versus complex), surgeon's experience of distal tibia fracture surgery (less than 15 cases versus more than 15 cases. The Paired t-test was used to determine if there were differences in the extent of usefulness of 3D printing in assessing fracture configuration and in preoperative planning and selection of the locking plate between simple and complex distal tibia fractures. The level of statistical significance was set at p < 0.05.

## 3. Results

A total of 102 orthopedic surgeons were interviewed. For the simple distal tibia fracture, 32 (31%) surgeons changed their selection of plate after using the 3D printed models, and for the complex distal tibia fracture the corresponding number was 56 (55%). More surgeons changed their selection of plate for the complex distal tibia fracture than the simple fracture (p < 0.001) (Table 1).

Forty-nine percent (28/57) of inexperienced surgeons (operating experience of less than 15 cases) and 9% (4/45) of experienced surgeons (operating experience of more than 15 cases) changed their selection of plate after using 3D printed models for the simple fracture, and the difference was significant (p < 0.001) (Table 2).

Seventy-four percent (42/57) and 31% (14/45) of inexperienced and experienced surgeons, respectively, changed their selection of plate for the complex fracture after using 3D printed models, and the difference was significant (p < 0.001) (Table 3).

Surgeons rated the extent of usefulness of the 3D models in obtaining fracture configuration as a mean of 4.32 ± 2.26 points on a 10-point NRS for the simple fracture and 5.75 ± 2.41 points for the complex fracture. The difference between the simple and complex fractures was significant (p < 0.001). Surgeons rated the extent of usefulness of the 3D models in preoperative planning and selection of the locking plate as a mean of 4.84 ± 2.54 points for the simple fracture and 6.63 ± 2.54 points for the complex fracture. The difference between the simple and complex fractures was significant (p < 0.001). For the simple distal tibia fracture, 51 (50%) surgeons responded that they would use 3D models in their practice if such models were provided, whereas for the complex distal tibia fracture the corresponding number was 68 (67%). More surgeons would use 3D models in their practice for complex fractures than for simple fractures (p < 0.001). Seventy-five percent (43/57) of inexperienced surgeons and 18% (18/45) of experienced surgeons responded that they would use 3D models in their practice for simple fractures, and the difference was significant (p < 0.001). Eighty-six percent (49/57) of inexperienced surgeons and 43% (19/45) of experienced surgeons responded that they would use 3D models in their practice for complex fractures, and the difference was significant (p < 0.001).

## 4. Discussion

This study investigated the surgeons' opinions about and need for 3D printing in fracture surgery. The most important finding was that a large fraction of orthopedic surgeons changed their selection of plate after using 3D printed models for simple and complex distal tibia fractures (31% and 55%, respectively). With regard to surgeon's experience and complexity of fracture, plate selection was most frequently changed by inexperienced surgeons for the complex fracture (74%) and least frequently by experienced surgeons for the simple fracture (9%). Many orthopedic surgeons responded that they would use 3D printed models in their practice (50% for the simple fracture and 67% for the complex fracture). Inexperienced surgeons responded that they would use 3D models for complex fractures especially often (86%). In contrast, only 18% of experienced surgeons found 3D printed models useful for simple fractures.

3D printing in the field of fracture surgery may eventually have a niche similar to that of CT performed for evaluation of fracture. CT scans are useful for evaluation of complex fractures and intra-articular fractures because they can provide information that cannot be obtained by simple X-ray examination. However, surgeons do not routinely request CT scans for all distal tibia fractures because radiography is typically sufficient for determining fracture configuration. In the current study, 82% of experienced surgeons were reluctant to use 3D printing for simple distal tibia fracture even when 3D models were provided. In contrast, for complex fracture, 86% of inexperienced surgeons wanted to use 3D printed models. Surgeons rated the extent of usefulness of 3D models in determining fracture configuration as a mean of 5.75 ± 2.41 points on a 10-point NRS for the complex fracture, which was significantly higher than the value for the simple fracture (p < 0.001). Similar to CT, 3D printing will be more useful for complex fractures than for simple fractures.

However, even for simple fractures, 49% of inexperienced surgeons changed their selection of plate after using 3D models, and 75% of inexperienced surgeons wanted to use 3D models in their practice. This shows that, even for simple fractures, 3D models can be useful for surgeons who are still learning distal tibia fracture surgery and have not reached the plateau of the learning curve. Considerations of patient safety and emphasis on operating room efficiency restrict the possibilities to gain initial experience by performing surgeries in the operating room [19, 20]. Training can be safely performed in a low-stress environment using cadavers and synthetic bone [21–27]. However, neither of these two substitutes can fully imitate fractured bone that the surgeon will eventually operate on. Fracture configuration can vary depending on the case, and every complex fracture can appear unique for a beginner until sufficient experience is gained. Therefore, even when surgical simulation training on cadavers or synthetic bone is over, the opportunity to visualize and handle a replica of the fractured bone and fit the implants before surgery can be enormously helpful in preoperative planning and in building confidence [22, 24].

It has been suggested that 3D printing can be the future of fracture surgery, but we know of no studies of surgeons' needs and views on the use of 3D printed models in fracture surgery [15]. Based on the results of the current study, 3D models should be primarily used for training, especially for complex fractures. In terms of technological development of 3D printing, the focus should be on how to imitate the texture of soft tissues, such as skin, muscle, tendons and ligaments, and cortical and cancellous bone to allow beginners perform simulation surgery on 3D models that are as similar as possible to human tissues [28]. Three-dimensional models may not be necessary for experienced surgeons, especially in the case of simple fractures. However, they can still be useful for familiarization with new implants or procedures.

This study has some limitations. The fact that many surgeons changed their selection of plate after using 3D models and wanted to use them in their practice may not be sufficient to conclude that 3D printing is an effective tool. For example, surgeons and patients may be reluctant to use 3D models if the cost is too high. Furthermore, surgeons have an opportunity to change their choice of plate during a real surgery by placing the plate after fracture reduction. The 3D models only represented the fractured tibia and the tibia before injury modeled by mirror imaging of the tibia on the opposite side. The tibia after fracture reduction is different from the tibia before injury unless a perfect anatomical reduction is achieved. However, we believe that the use of 3D printing for selecting the implant is still feasible. In real practice, it is difficult and inefficient to sterilize all sets of instruments for all different locking plates and to apply each of them to the fractured tibia to choose the one with the best fit. Instead, most surgeons select one plate that they believe to be the best choice before the surgery. Unlike experienced surgeons, beginners may have difficulties selecting the plate in this manner. Although the plate selected by using 3D models may not be the best choice, it will likely be a better choice than plates selected based on X-ray and CT images. Furthermore, using 3D printed models will result in more time spent on assessing the fracture configuration and planning the operation. Preoperative planning is recognized as an essential prerequisite for successful surgery in complicated cases [29, 30]. This aspect is especially important for beginners. The main problems may be the cost and time required for the production of 3D models. It remains to be determined whether the benefits of this technique justify the increased cost. However, the technical developments and increasing popularity of 3D printing are expected to lower the cost and time required to produce 3D models for fracture surgery.

The validity of division of surgeons on experienced and inexperienced using a threshold of 15 cases may be a matter of debate. The learning curve for distal tibia fracture surgery has not been thoroughly studied. For proximal femoral nailing, operative speed after 15 surgeries did not significantly differ from that of more experienced surgeons [31]. For pediatric supracondylar humerus fractures, 15 cases were assumed to be required to reach the plateau of the learning curve [32]. In the initial stage of this study, we asked professors at university hospitals how many distal tibia fracture cases they handled. Even though they could not provide an exact number, they were sure that the number exceeded 15 cases. Residents kept an exact count, and the number of cases was less than 15.

## 5. Conclusion

In conclusion, the use of real-size 3D-printed models often changed surgeon's preoperative selection of locking plates, especially when inexperienced surgeons evaluated complex fractures. However, for experienced surgeons and simple fractures, the use of 3D models was not very beneficial, and experienced surgeons did not generally consider 3D printing a technique they would want to utilize in surgical practice. Future applications of 3D models should focus on training beginners in fracture surgery, especially when complex fractures are concerned.

## Figures and Tables

**Table 1:** Change of plate selection after using real-size 3D printed models.

	Changed numbers (%)	Not changed numbers (%)	Total numbers
Simple distal tibia fracture	32 (31%)	70 (69%)	102
Complex distal tibia fracture	56 (55%)	46 (45%)	102

**Table 2:** Change of plate selection for simple distal tibia fracture after using 3D models.

	Changed numbers (%)	Not changed numbers (%)	Total numbers
Inexperienced group	28 (49%)	29 (51%)	57
Experienced group	4 (9%)	41 (91%)	45

Inexperienced group: orthopedic surgeons who have operated less than 15 cases of distal tibia fracture.

Experienced group: orthopedic surgeons who have operated more than 15 cases of distal tibia fracture.

**Table 3:** Change of plate selection for complex distal tibia fracture after using 3D models.

	Changed numbers (%)	Not changed numbers (%)	Total numbers
Inexperienced group	42 (74%)	15 (26%)	57
Experienced group	14 (31%)	31 (69%)	45

Inexperienced group: orthopedic surgeons who have operated less than 15 cases of distal tibia fracture.

Experienced group: orthopedic surgeons who have operated more than 15 cases of distal tibia fracture.

## Data Availability

The data used to support the findings of this study are available from the corresponding author upon request.

## Supplementary Materials

Supplementary Materials
* File 1*: survey sheet.
