# Supplementary material for: Can Preoperative 3D Printing Change Surgeon's Operative Plan for Distal Tibia Fracture?
Source: Biomed Res Int. 2019 Feb 11;2019:7059413. doi: 10.1155/2019/7059413 (PMC6388342; doi:10.1155/2019/7059413)
Supplement: Supplementary Materials — File 1: survey sheet. [file 7059413.f1.doc]

**Survey Sheet**

**Thank you very much for agreeing to participate in the study.**

Please check “V” where appropriate.

I am an orthopedic surgeon working at ① University hospital as a professor or consultant

② Local hospital as an orthopedic board certified specialist, or clinical fellow, ③ I am an orthopedic resident.

I have operated (① more than 15 cases, ② less than 15 cases) of distal tibia fracture as the operating surgeon.

**Q1.** Please examine X-rays (AP, lateral, mortise views) and CT (sagittal, coronal, axial, and 3D reconstruction images) images of the two distal tibia fracture on the tablet PC

Which Anatomic contoured Locking plate would you use for each of the two cases?

① Zimmer Periarticular Distal Medial Tibial Locking Plate (ZPLP; Zimmer, Warsaw, IN, USA)

② Zimmer Periarticular Distal Lateral Tibial Locking Plate (ZPLP; Zimmer, Warsaw, IN, USA)

③ LCP Metaphyseal Plate for distal medial tibia (Synthes, Oberdoft, Switzerland),

④ LCP Low Bend Medial Distal Tibia Plates 3.5 mm (Synthes, Oberdoft, Switzerland),

⑤ AxSOS Distal Medial Tibial Plate (Stryker, Mahwah, NJ)

⑥ Implant not present in the picture

Please check “V” for each of the two cases

**Case 1**

①, ②, ③, ④, ⑤, ⑥

**Case 2**

①, ②, ③, ④, ⑤, ⑥

Please examine the real-size 3D models of the fractured tibia and the normal tibia created by mirroring the tibia on the unaffected side. You may study the fracture configuration and simulate the placement of the plates on the fractured tibia considering screw trajectories in the plate for fixation of fracture fragments.

**Q2.** Which Anatomic contoured Locking plate would you use for each of the two cases?

**Case 1**

①, ②, ③, ④, ⑤, ⑥

**Case 2**

①, ②, ③, ④, ⑤, ⑥

**Q3.** How useful was 3D printing in evaluating fracture configuration? Please check “V” at the number for each of the two cases.

Not useful at all **Case 1** extremely useful

0 1 2 3 4 5 6 7 8 9 10

**Case 2**

0 1 2 3 4 5 6 7 8 9 10

**Q4.** How useful was 3D printing in preoperative planning and selection of the locking plate? Please check “V” at the number for each of the two cases.

Not useful at all **Case 1** extremely useful

0 1 2 3 4 5 6 7 8 9 10

**Case 2**

0 1 2 3 4 5 6 7 8 9 10

**Q5**. Would you use 3D models in your practice for each of the two distal tibia fractures if such models are available?

**Case 1**

1. Yes ② No

**Case 2**

1. Yes ② No

**Thank you very much for taking your precious time for the study.**
